# Effects of the Lower Irrigation Limit and Drip Irrigation Flow Rate on Root Traits and Bacterial Community Structure of Tomatoes Grown in Coconut Coir

**DOI:** 10.3390/plants15172672

**Published:** 2026-08-31

**Authors:** Mengfan Liu, Xiaokun Feng, Haitao Wang, Zhanming Tan, Xiaoyang Liang, Qi Wang, Yujie Wang, Jiandong Wang

**Affiliations:** 1 College of Horticulture and Forestry, Tarim University, Alar 843302, China; 2 Institute of Environment and Sustainable Development in Agriculture, Chinese Academy of Agricultural Sciences, Beijing 100081, China; 3 College of Life Sciences and Food Engineering, Hebei University of Engineering, Handan 056038, China; 4 Department of Bioresource Engineering, McGill University, Montreal, QC H9X 3V9, Canada

**Keywords:** lower irrigation limit, drip irrigation flow rate, coir substrate, microbial community, co-occurrence network

## Abstract

Water management is a key factor affecting crop physiology and rhizosphere microbial ecology in protected agriculture. However, the responses of root development and bacterial community structure to different lower irrigation limits and drip irrigation flow rates in coir substrates remain poorly understood. This study investigated greenhouse-grown tomatoes in the Gobi region under three lower irrigation limits (30%, 50%, and 70% of substrate water-holding capacity) and two drip irrigation flow rates (1 and 2 L·h^−1^). Substrate moisture, root traits, bacterial community structure, co-occurrence networks, and functional potential were evaluated. Increasing the lower irrigation limit maintained higher and more stable substrate moisture and generally enhanced root length, root surface area, root tip number, and root activity. W3Q1 promoted greater spatial expansion of the root system, whereas W2Q2 produced the largest root volume and maintained relatively high root activity. Irrigation combination significantly altered bacterial community structure but had limited effects on bacterial richness. W2Q1 enriched Bacillota-related taxa, whereas W2Q2 enriched Pseudomonadota and exhibited greater bacterial network connectivity and modularity. FAPROTAX-based functional prediction indicated that the bacterial communities were mainly associated with chemoheterotrophy, organic matter decomposition, and nitrogen transformation. Overall, the lower irrigation limit primarily regulated substrate moisture and root traits, whereas the effects of drip irrigation flow rate were trait- and irrigation-limit-dependent, with significant main and interaction effects on root volume. These findings provide a basis for optimizing precision irrigation in coir-substrate tomato cultivation in arid greenhouse systems.

## 1. Introduction

The Gobi region is characterized by abundant solar and thermal resources but scarce precipitation, intense evaporation, and naturally infertile soils, which severely constrain the development of conventional agriculture [1]. Solar greenhouses combined with soilless cultivation provide an important production system for Gobi agriculture, improving water use efficiency while mitigating soil degradation and continuous cropping obstacles [2,3]. Compared with soil, coconut coir has distinct pore structures and water transport characteristics. Moreover, the restricted root-zone volume in substrate cultivation makes root-zone water status and the rhizosphere environment particularly sensitive to irrigation management [4,5]. Therefore, the relationships among water availability, root development, and microbial communities established in soil-based systems may not be directly applicable to coconut coir cultivation. Elucidating changes in root-zone water status under different lower irrigation limits and their relationships with root growth and microbial communities is therefore essential for optimizing root-zone water management in soilless cultivation systems in Gobi greenhouses. Previous studies have demonstrated that irrigation regimes can significantly affect crop growth, root development, and rhizosphere microbial communities [6,7,8,9,10,11]. As a critical interface connecting plants with the rhizosphere environment, roots may be closely associated with changes in rhizosphere microbial communities through alterations in root morphology and root exudation, which affect root–substrate contact area, the spatial range of resource acquisition, and carbon supply [12,13,14,15]. Meanwhile, irrigation can directly modify substrate water content, aeration, and nutrient transport. Thus, the responses of roots and microorganisms to irrigation are closely linked through interconnected ecological processes within the rhizosphere.

The lower irrigation limit, as a key threshold determining irrigation initiation, directly regulates the dynamics of substrate water content [16]. A lower irrigation limit results in greater fluctuation in substrate water content and may reduce microbial diversity [17]. However, drought conditions may also select for drought-tolerant or plant growth-promoting microbial taxa, thereby modulating plant responses to environmental stress [18]. In contrast, a higher irrigation limit maintains a relatively moist substrate environment that favors the survival of most microorganisms. Nevertheless, excessively wet conditions may promote the proliferation of soil-borne pathogens while weakening the effects of antagonistic microorganisms, thereby aggravating root rot [19]. In addition, drip irrigation flow rates can produce different wetting patterns depending on the initial water content of the substrate, altering the spatial distribution of water and consequently affecting oxygen diffusion and water availability in the rhizosphere [20]. Previous studies have shown that low drip irrigation flow rates can produce smaller and more uniformly distributed wetted zones, potentially facilitating broader root distribution and interaction with diverse microbial communities. In contrast, high drip irrigation flow rates may create more concentrated wetted zones and induce localized oxygen deficiency in the rhizosphere, thereby altering the ecological niches of microbial communities [21]. Although numerous studies have investigated the effects of irrigation level, water status, or irrigation frequency on root systems and microbial communities, most have focused on soil-based cultivation systems. By comparison, the responses of the root-zone environment, root development, and microbial communities to different lower irrigation limits and drip irrigation flow rates in coconut coir cultivation systems in Gobi greenhouses remain poorly understood. In particular, the coordinated responses of the coconut coir root-zone environment, root development, bacterial community structure, and co-occurrence networks to combinations of lower irrigation limits and drip irrigation flow rates have yet to be systematically elucidated.

Accordingly, this study investigated tomato plants cultivated in coconut coir substrate in a Gobi greenhouse in Xinjiang, China, under a combination of different lower irrigation limits and drip irrigation flow rates. An integrated analysis was conducted at three levels—the substrate environment, root growth, and rhizosphere bacterial community—to (1) determine the effects of different lower irrigation limits and drip irrigation flow rates on coconut coir substrate temperature and water content as well as tomato root characteristics; (2) elucidate the responses of microbial diversity, community structure, differentially abundant bacterial taxa, and predicted microbial functions to different water management regimes; and (3) construct microbial co-occurrence networks and evaluate how irrigation management alters potential bacterial association patter and network topology. Unlike previous studies that have primarily examined the separate effects of irrigation on either root systems or microbial communities, this study focuses on the responses and interrelationships among the root-zone environment, root development, and bacterial communities under the combined regulation of lower irrigation limits and drip irrigation flow rates within the unique root-zone environment of coconut coir cultivation in Gobi greenhouses. The findings are expected to provide an ecological basis for precision irrigation management in soilless cultivation systems in Gobi greenhouses.

## 2. Results

### 2.1. Substrate Environment

**Figure 1:**
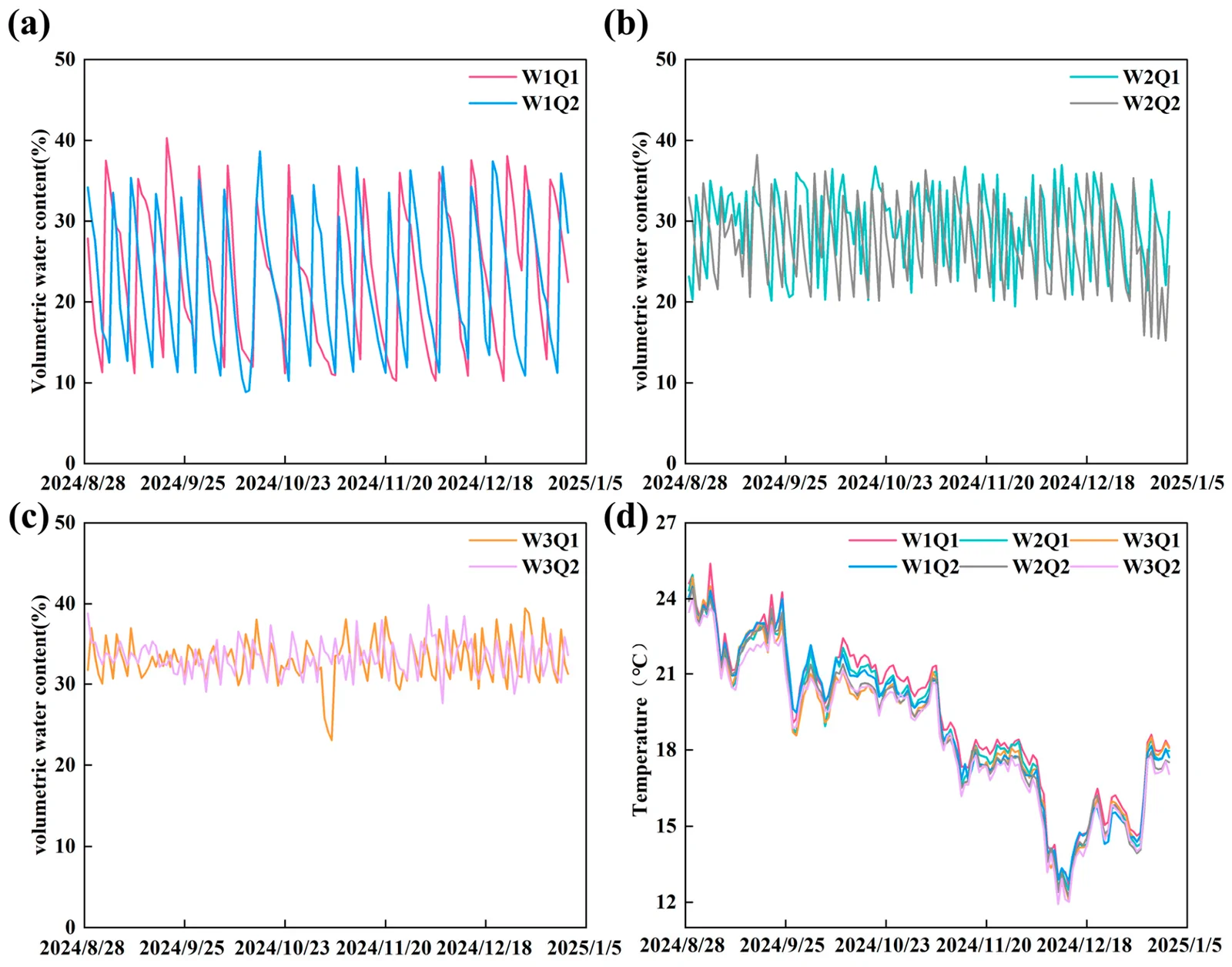
Temporal variation in daily mean substrate volumetric water content and temperature during the experimental period. (**a**–**c**) Daily mean substrate volumetric water content under W1, W2, and W3, respectively, with Q1 and Q2 representing the two drip irrigation flow rates; (**d**) daily mean temperature under the six irrigation treatments.

The dynamic effects of different combinations of lower irrigation limits and drip irrigation flow rates on the daily mean volumetric water content and temperature of the coir substrate at different tomato growth stages are shown in Figure 1. Throughout the entire growth period, the volumetric water content exhibited distinct temporal variation patterns among treatments. The volumetric water content of the substrate fluctuated between approximately 11% and 39%, showing periodic sawtooth-like variation in response to irrigation and water consumption. Substrate volumetric water content was primarily regulated by the lower irrigation limit. Under the high lower irrigation limit treatment (W3), the volumetric water content remained consistently high, ranging from 29.6% to 37.4%, whereas under the low lower irrigation limit treatment (W1), it exhibited a wider fluctuation range of 10.3–36.2%. The W3Q2 treatment showed the highest frequency of volumetric water content fluctuation (43 times), which was significantly higher than that observed under the W1Q1 treatment (22 times). Substrate temperature varied dynamically with the progression of tomato growth stages and exhibited certain stage-specific fluctuations. At the beginning of the observation period, substrate temperature was approximately 18–26 °C and subsequently showed an overall decreasing trend. From October to November, it was mainly maintained at approximately 16–23 °C, followed by a further decline in December, reaching its lowest level toward the end of the observation period, with a minimum of approximately 13.6 °C. The overall temperature variation trends were similar among treatments. The W1Q1 treatment generally exhibited relatively higher temperatures during most periods, whereas the W3Q2 treatment showed relatively lower temperatures. In addition, temperatures under the Q1 treatments were generally slightly higher than those under the corresponding Q2 treatments. These results indicate that the different treatments altered the periodic fluctuation characteristics of substrate water content and the temperature level, but did not change the overall seasonal variation trend in substrate temperature.

### 2.2. Root Traits and Root Activity

**Figure 2:**
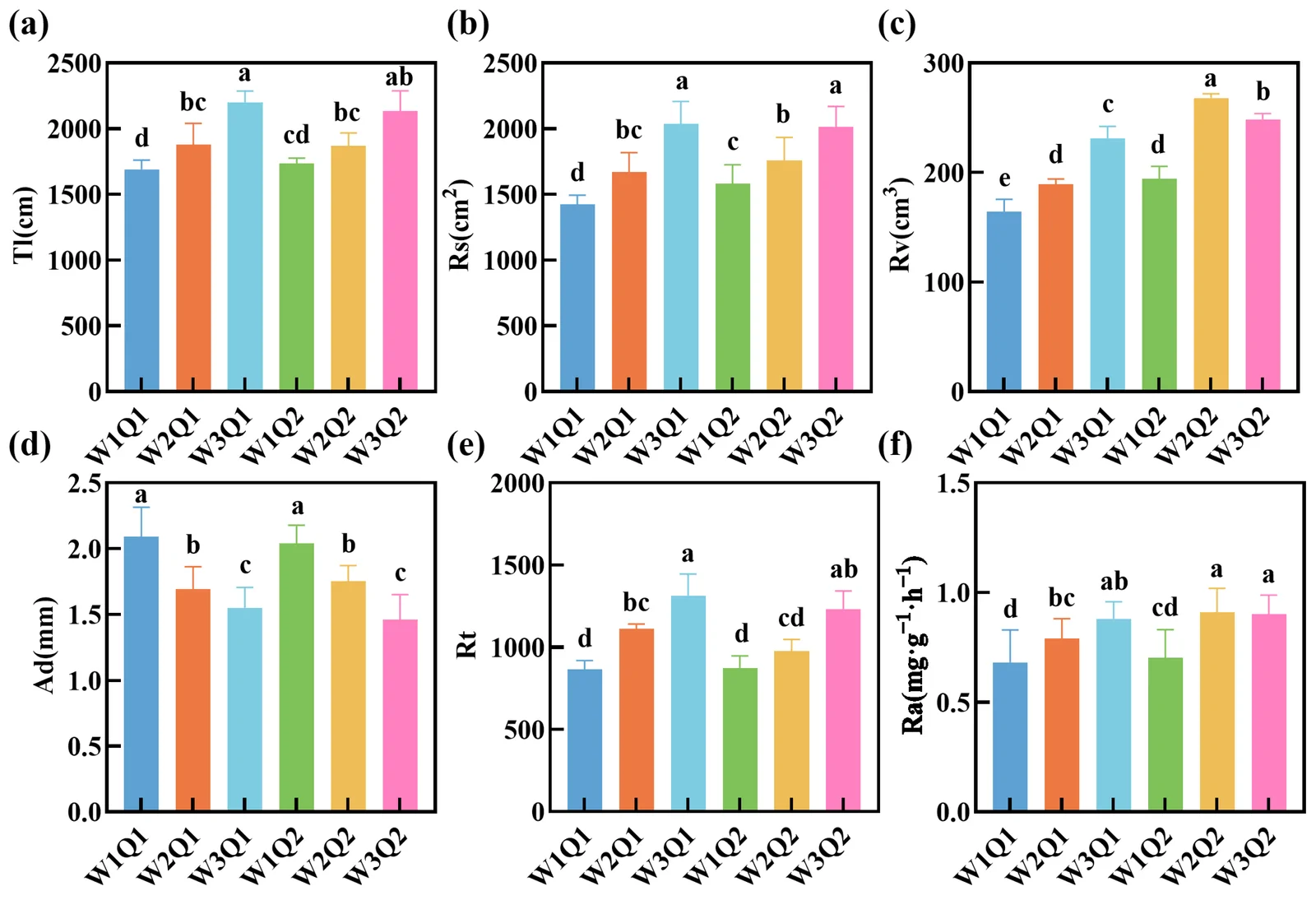
Tomato root traits and root activity under different combinations of lower irrigation limit and drip irrigation flow rate. (**a**) Tl, total root length (cm); (**b**) Rs, total root surface area (cm^2^); (**c**) Rv, total root volume (cm^3^); (**d**) Ad, mean root diameter (mm); (**e**) Rt, number of root tips; and (**f**) Ra, root activity (mg·g^−1^·h^−1^). Data are presented as the mean ± standard deviation (SD; *n* = 3). Different lowercase letters above the bars indicate significant differences among treatments under the same cultivation conditions, as determined by one-way analysis of variance followed by Duncan’s multiple range test (*p* < 0.05).

Tomato root morphology and root activity differed markedly among the combination of lower irrigation limit and drip irrigation flow rate (Figure 2). Overall, as the lower irrigation limit increased, total root length (Tl), root surface area (Rs), root volume (Rv), number of root tips (Rt), and root activity (Ra) generally increased, whereas average root diameter (Ad) decreased. Two-way ANOVA showed that the lower irrigation limit significantly affected all measured root traits. Drip irrigation flow rate and its interaction with the lower irrigation limit significantly affected only root volume, with no significant effects on the other root traits (Table 1). The effects of drip irrigation flow rate on these root traits varied depending on the lower irrigation limit. Among the root morphological traits, W3Q1 produced the greatest total root length, root surface area, and number of root tips. Total root length under W3Q1 reached 2201.28 cm, which was significantly higher than that under W1Q1 and W2Q1 by 30.2% and 17.1%, respectively. Root surface area showed a pattern similar to that of total root length. The root surface areas under W3Q1 and W3Q2 were 2036.26 and 2013.48 cm^2^, respectively, both of which were significantly greater than those under the other treatments. In particular, W3Q1 increased root surface area by 43.0% compared with W1Q1, demonstrating a clear advantage. The greatest root volume was observed under W2Q2, reaching 268.23 cm^3^, which was significantly higher than that under the other treatments, followed by W3Q2 at 248.39 cm^3^. Average root diameter decreased as the lower irrigation limit increased. The highest values were observed under W1Q1 and W1Q2, at 2.09 and 2.04 mm, respectively, whereas the lowest value occurred under W3Q2, at only 1.46 mm, representing a 30.1% reduction relative to W1Q1. Root activity was highest under W2Q2 and W3Q2, at approximately 0.91 and 0.90 mg·g^−1^·h^−1^, respectively. These values did not differ significantly from those under W3Q1 (0.88 mg·g^−1^·h^−1^) but were significantly higher than those under W1Q1 (0.68 mg·g^−1^·h^−1^). Overall, increasing the lower irrigation limit favored root elongation, expansion of root surface area, and root tip formation while reducing average root diameter. Among the treatments, W3Q1 was more favorable for the spatial expansion of the root system, whereas W2Q2 was more conducive to increasing root volume and maintaining high root activity.

### 2.3. Substrate Bacterial Community

#### 2.3.1. Composition of the Substrate Bacterial Community

**Figure 3:**
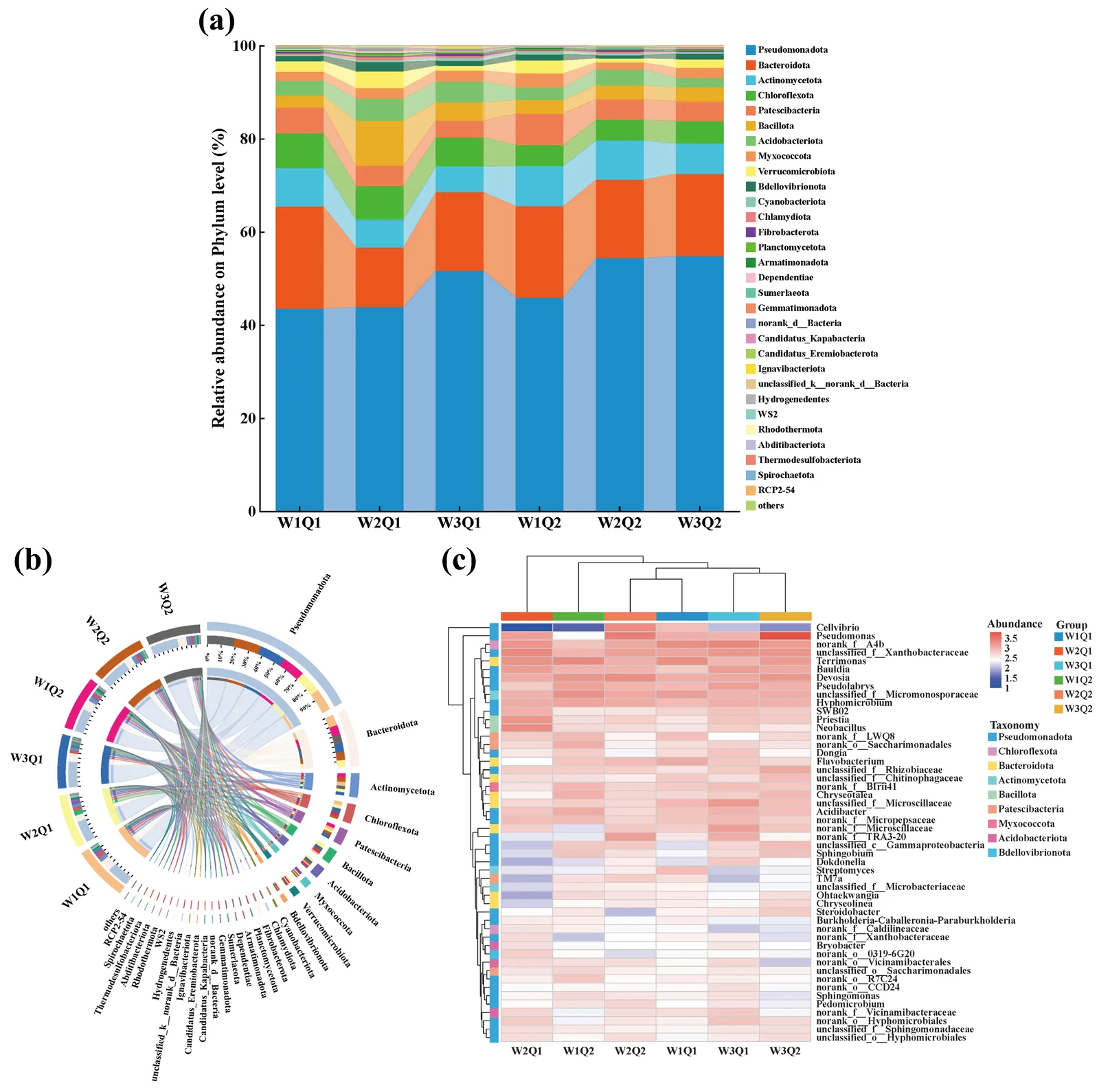
Substrate bacterial community composition. (**a**) Relative abundances of bacterial phyla across treatments; (**b**) detailed distribution of the dominant bacterial taxa at the phylum level; and (**c**) relative abundances of bacterial genera across all samples.

The composition of the tomato rhizosphere bacterial communities at the phylum and genus levels under different combinations of lower irrigation limits and drip irrigation flow rates is shown in Figure 3. Clear differences in bacterial community composition were observed among treatments. At the phylum level (Figure 3a), Pseudomonadota, Bacteroidota, Actinomycetota, Chloroflexota, and Patescibacteria were the dominant bacterial phyla, collectively accounting for more than 80% of the total relative abundance. The relative abundance of Bacillota was markedly higher under W2Q1, whereas Chloroflexota accounted for relatively larger proportions under W1Q1 and W2Q1. The chord diagram further illustrated the association between the different treatments and the major bacterial taxa (Figure 3b). Pseudomonadota showed strong association with all treatments, whereas the distribution pattern of the other dominant phyla varied among treatments. W2Q1 was more strongly associated with Bacillota, while W2Q2 and W3Q2 showed stronger association with Pseudomonadota, consistent with the phylum-level relative-abundance results. The genus-level heatmap further revealed the distribution of dominant genera and the similarities among samples (Figure 3c). *Cellvibrio*, *Pseudomonas*, *Terrimonas*, *Devosia*, *Pseudolabrys*, *Hyphomicrobium*, *Flavobacterium*, *Sphingobium*, *Streptomyces*, and *Sphingomonas* were among the predominant genera. Hierarchical clustering indicated that the bacterial community composition under W1Q1 and W3Q1 was relatively similar. W1Q2 and W2Q2 also exhibited a certain degree of similarity, whereas W2Q1 showed a comparatively distinct genus-level community composition from the other treatments. These results demonstrate that the different irrigation treatments altered both the composition of dominant bacterial taxa and their relative-abundance distribution in the substrate.

#### 2.3.2. Substrate Bacterial α and β-Diversity

**Figure 4:**
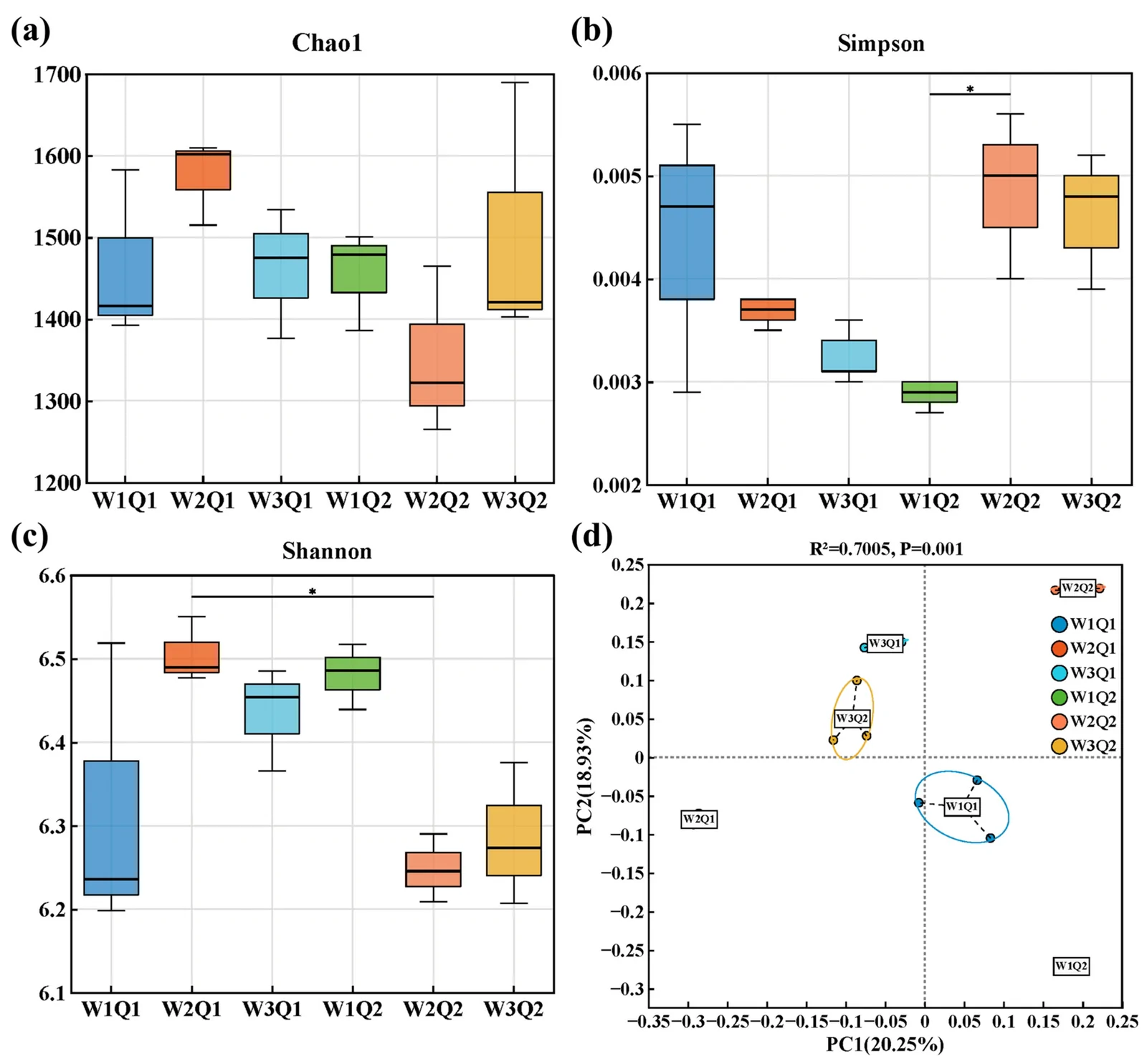
Alpha and beta diversity of the substrate bacterial community. (**a**–**c**) Alpha-diversity indices of the substrate bacterial community under different combinations of lower irrigation limit and drip irrigation flow rate, including the Chao1 index, Simpson dominance index (D), and Shannon index; (**d**) principal coordinate analysis (PCoA) based on Bray–Curtis distances, showing differences in bacterial community structure among treatments. For the Simpson dominance index (D), lower values indicate lower community dominance and higher diversity. * indicates a significant difference between the connected treatments at *p* < 0.05.

The α- and β-diversity of bacterial communities under different combinations of lower irrigation limit and drip irrigation flow rate are shown in Figure 4. The Chao1 index (Figure 4a) showed some variation in bacterial community richness among treatments. W2Q1 had the highest Chao1 index (1601), whereas W2Q2 had the lowest value (1322), with the remaining treatments showing intermediate values. Two-way ANOVA showed no significant effects of the lower irrigation limit, drip irrigation flow rate, or their interaction on the Chao1 index (*p* = 0.909, 0.202, and 0.086, respectively). The Simpson dominance index (D) (Figure 4b) also varied among treatments. The Simpson dominance index under W2Q2 was significantly higher than that under W1Q2 (*p* < 0.05). W1Q2 had the lowest Simpson dominance index (D; 0.0029), indicating lower community dominance and higher diversity, whereas W3Q2 exhibited a relatively high value (0.0048), indicating higher community dominance and relatively lower diversity. The Shannon index showed a somewhat different pattern from the Simpson dominance index (D) (Figure 4c). W2Q1 had the highest Shannon index (6.49), followed by W1Q2 and W3Q1, whereas W1Q1, W2Q2, and W3Q2 had relatively lower values. In particular, the Shannon index under W2Q1 was significantly higher than that under W2Q2 (*p* < 0.05), indicating a treatment-specific response of bacterial community diversity to drip irrigation flow rate at the 50% lower irrigation limit. This result was consistent with the significant W × Q interaction detected by two-way ANOVA. Two-way ANOVA further showed that neither the lower irrigation limit nor drip irrigation flow rate significantly affected the Simpson dominance index (D) or Shannon index (*p* > 0.05), whereas their interaction was highly significant for both indices (*p* < 0.01; Table 1). These results indicate that the effect of drip irrigation flow rate on bacterial community diversity depended on the level of the lower irrigation limit. Principal coordinate analysis (PCoA) based on Bray–Curtis distances revealed a clear separation of bacterial community structures among different combinations of lower irrigation limit and drip irrigation flow rate (Figure 4d). The first two axes explained 39.18% of the total variation, with PCoA1 and PCoA2 accounting for 20.25% and 18.93%, respectively. Samples within the same treatment generally clustered closely, indicating good within-treatment reproducibility, whereas samples from different treatments were clearly separated in the ordination space, suggesting that different lower irrigation limits and drip irrigation flow rates markedly altered bacterial community structure. PERMANOVA further confirmed significant differences in bacterial community composition among treatments (R^2^ = 0.7005, *p* = 0.001).

#### 2.3.3. Differential Taxa in the Substrate Bacterial Community

**Figure 5:**
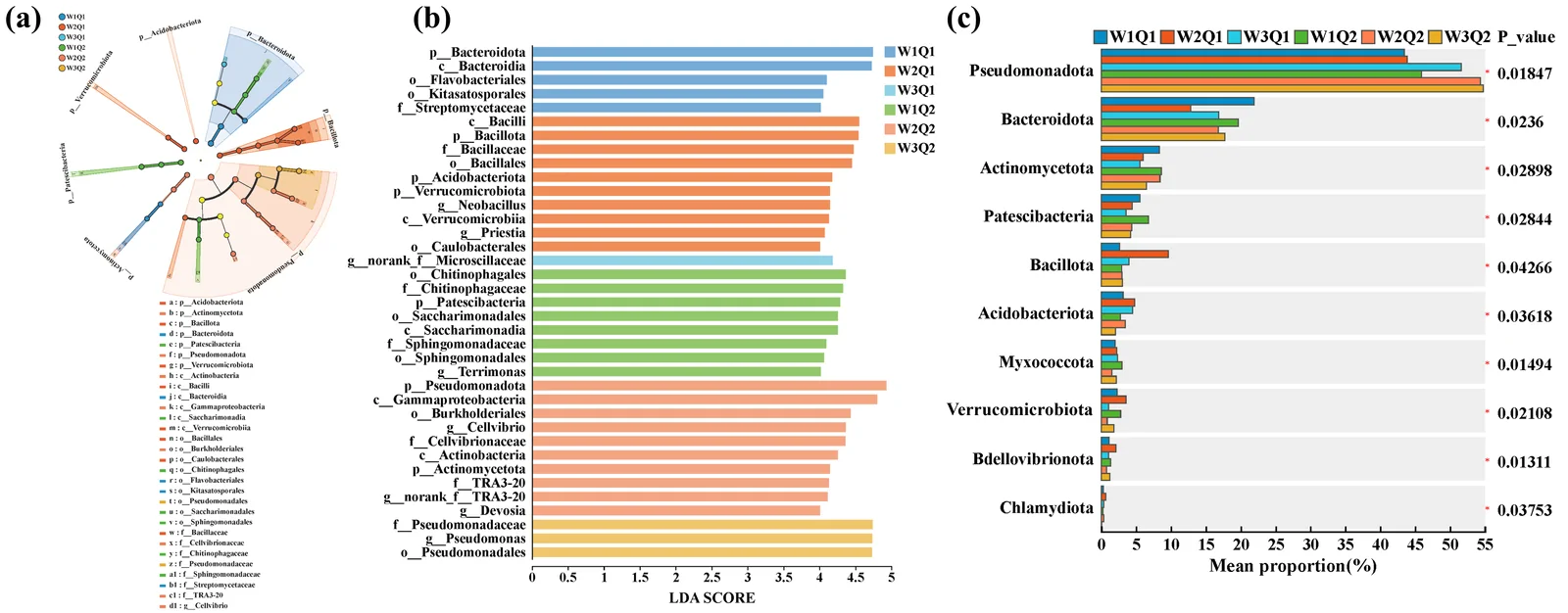
Differences in the substrate bacterial community. (**a**) LEfSe-based cladogram of differentially abundant taxa; (**b**) distribution of linear discriminant analysis (LDA) scores for differentially abundant taxa; and (**c**) comparison of the relative abundances of key taxa among treatments. * indicates significant differences among treatments at *p* < 0.05.

Differentially abundant bacterial taxa among treatments were identified using linear discriminant analysis effect size (LEfSe), with an LDA threshold of >4 (Figure 5). The LEfSe cladogram (Figure 5a) revealed distinct biomarker taxa under the different treatments. Specifically, the taxa enriched under W1Q1 mainly included Kitasatosporales, Streptomycetaceae, Bacteroidota, and the subordinate taxa Bacteroidia and Flavobacteriales. W2Q1 was characterized by the enrichment of Bacillota, Bacilli, Bacillales, Bacillaceae, Acidobacteriota, Verrucomicrobiota, *Bacillus*, *Neobacillus*, and *Priestia*. The biomarker taxa associated with W1Q2 mainly included Patescibacteria, Saccharimonadia, Chitinophagales, Sphingomonadales, Sphingomonadaceae, and *Terrimonas*. W2Q2 was primarily enriched in Pseudomonadota, Gammaproteobacteria, Burkholderiales, Cellvibrionaceae, *Cellvibrio*, Actinomycetota, and *Devosia*. The differentially abundant taxa under W3Q1 mainly comprised unclassified taxa associated with Microscillaceae, whereas W3Q2 was characterized by Pseudomonadales, Pseudomonadaceae, and *Pseudomonas*. LDA further quantified the contribution of the key differential taxa to community differentiation among treatments (Figure 5b). The biomarker taxa enriched under the different treatments exerted varying effects on bacterial community differentiation. Several differentially abundant taxa under W2Q2 exhibited relatively high LDA effect sizes, with Pseudomonadota showing the highest LDA score. This finding indicates that the key taxa enriched under the combination of a 50% lower irrigation limit and a drip irrigation flow rate of 2 L·h^−1^ showed the largest discriminatory effect size among the detected biomarkers. By contrast, although the other treatments also exhibited treatment-specific dominant taxa, their LDA scores and relative contribution were generally lower. Phylum-level differential abundance analysis (Figure 5c) showed that the relative abundances of Pseudomonadota, Bacteroidota, Actinomycetota, Patescibacteria, Bacillota, Acidobacteriota, Myxococcota, Verrucomicrobiota, Bdellovibrionota, and Chlamydiota differed significantly among treatments (*p* < 0.05). Pseudomonadota was relatively more abundant under W2Q2 and W3Q2, Bacteroidota was more abundant under W1Q1, and Bacillota was markedly enriched under W2Q1. These results demonstrate that the different irrigation treatments significantly altered the dominant bacterial taxa and their relative abundance distribution in the substrate.

#### 2.3.4. Co-Occurrence Networks of Substrate Bacterial Communities

**Figure 6:**
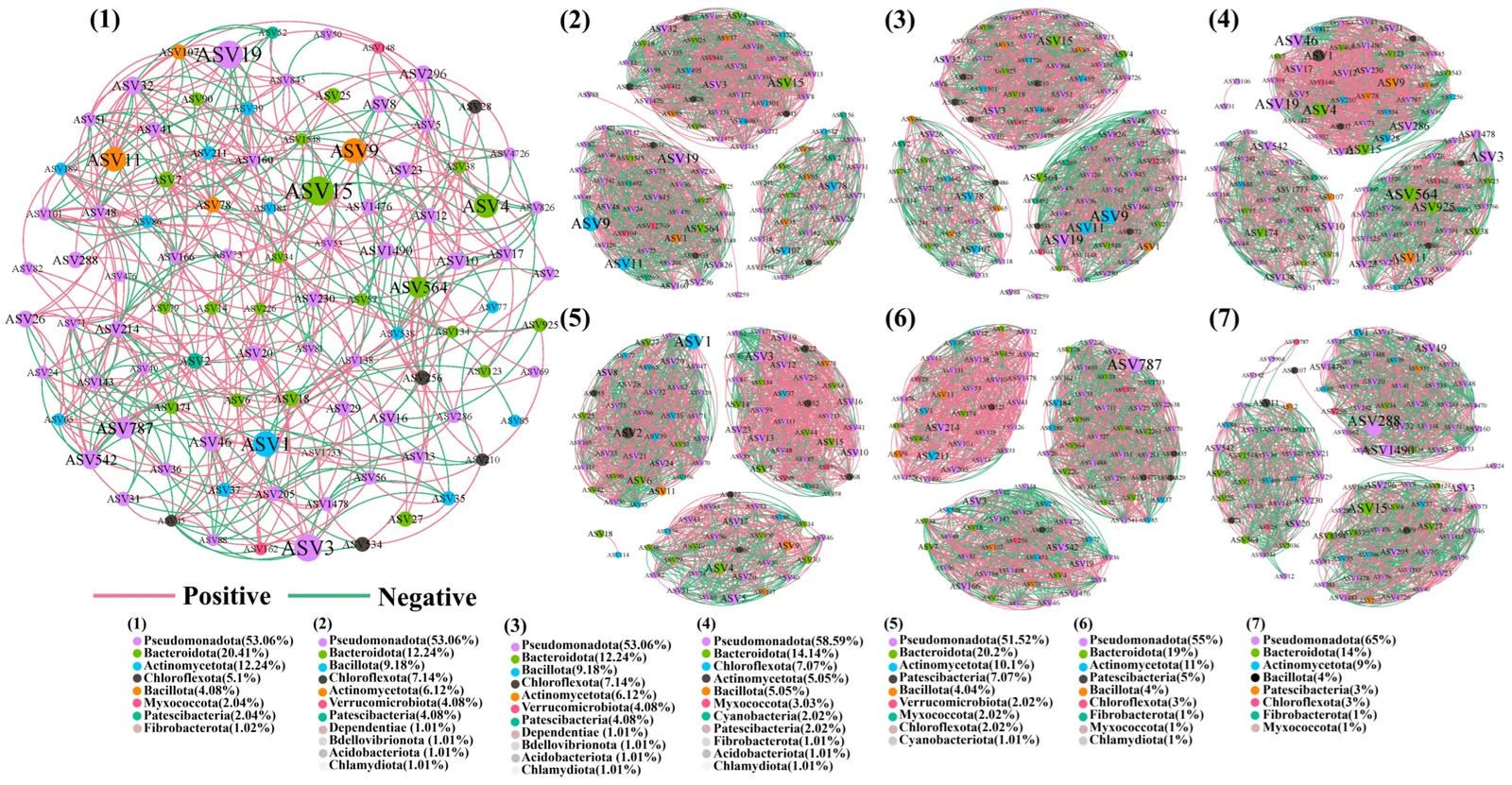
Co-occurrence network analysis of relationships among bacterial community ASVs. (**1**) Network constructed using samples from all treatments; (**2**–**7**) treatment-specific bacterial co-occurrence networks for W1Q1, W2Q1, W3Q1, W1Q2, W2Q2, and W3Q2, respectively. Networks were constructed based on Spearman correlation analysis (|r| ≥ 0.6, *p* < 0.05). Node colors represent bacterial taxa belonging to different phyla, and node sizes indicate taxon abundance. Pink and green edges represent positive and negative correlations between nodes, respectively.

Co-occurrence networks were constructed based on Spearman’s correlation analysis (|r| ≥ 0.6, *p* < 0.05) using the 100 most abundant nodes in each treatment (Figure 6). The overall network (Figure 6(1)), comprising the 98 most abundant nodes, contained 483 edges and had an average degree of 9.857, a network density of 0.102, and a modularity index of 0.489. Positive and negative correlations accounted for 59.42% and 40.58% of all associations, respectively. The number of nodes and edges, average degree, network density, and modularity coefficient varied among the different combinations of lower irrigation limits and drip irrigation flow rates (Table 2). Among the treatments, W2Q2 (Figure 6(6)) exhibited the largest number of edges (1622), the highest average degree (32.440), the greatest network density (0.328), and the highest modularity coefficient (0.662). The W3Q2 network (Figure 6(7)) had 1447 edges, an average degree of 28.940, and a network deity of 0.292, while retaining a relatively high modularity coefficient of 0.658. The modularity coefficients were relatively high across all treatments (0.582–0.662), indicating that the bacterial community networks exhibited a pronounced modular structure. Positive correlation accounted for 50.32–55.35% of the association in the treatment-specific networks, with W1Q2 showing the highest proportion of positive correlation, whereas negative correlation accounted for 44.65–49.68%. Overall, positive correlation slightly predominated across all treatment networks, indicating clear differences in the potential association pattern and network topological characteristics of substrate bacterial communities among irrigation treatments. Prominent node-associated taxa in the networks included Pseudomonadaceae and Bacteroidota, together with Bacillaceae, Actinomycetota, Chloroflexota, and Myxococcota. The distribution of these taxa across network nodes and the pattern of their connections differed visibly among treatments (Figure 6).

#### 2.3.5. Functional Prediction

**Figure 7:**
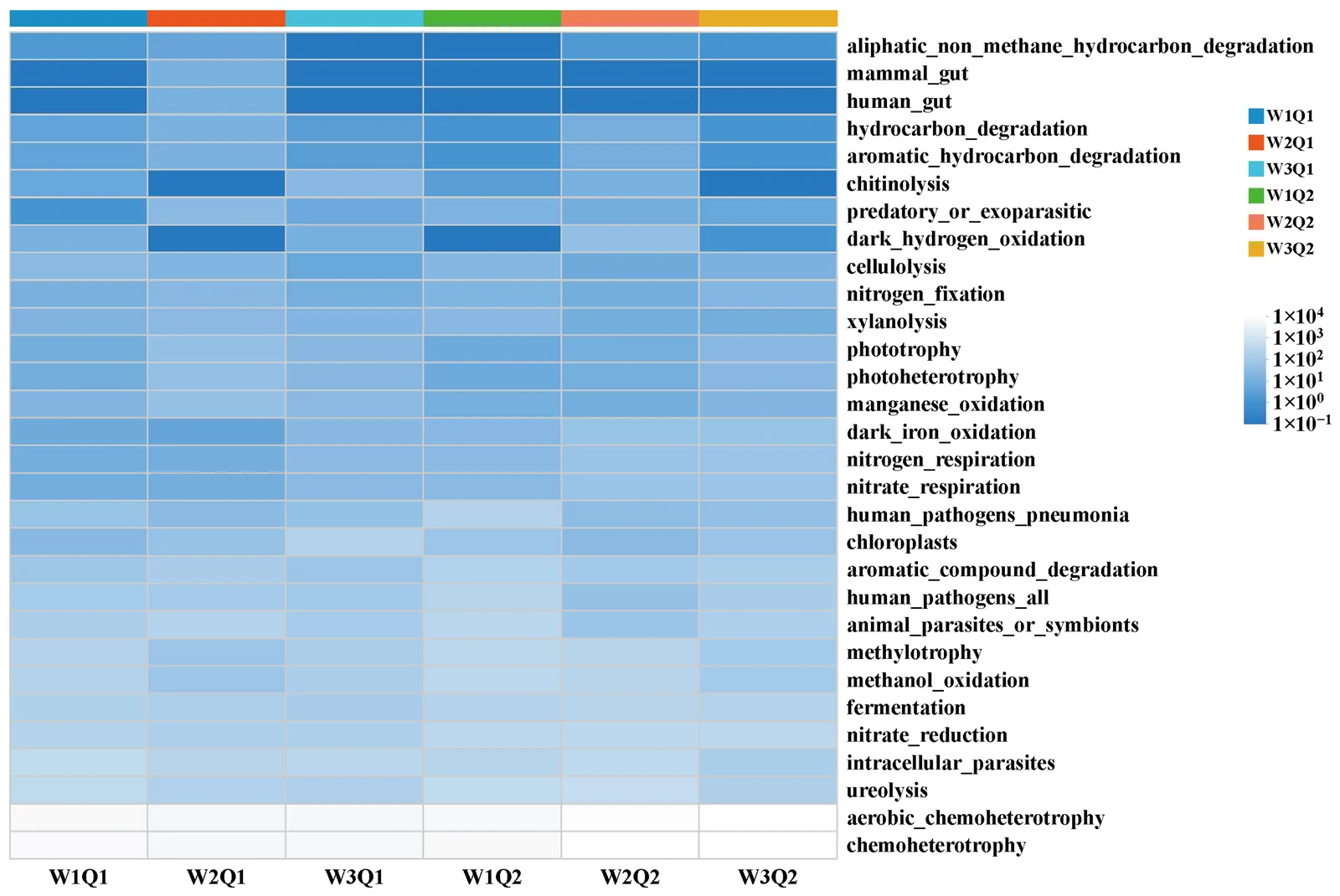
Functional prediction of the substrate bacterial community based on the FAPROTAX database.

Functional prediction based on the FAPROTAX database (Figure 7) showed that the overall metabolic functional profiles of the microbial communities were similar across different combinations of lower irrigation limit and drip irrigation flow rates. The predicted functions were mainly associated with heterotrophic metabolism, organic matter degradation, nitrogen cycling, and inorganic-element transformation. Chemoheterotrophy and aerobic chemoheterotrophy were the most abundant predicted functional categories across all treatments, indicating that the metabolic potential of the substrate bacterial communities was primarily related to organic carbon utilization. Other carbon cycle-related functions included aliphatic non-methane hydrocarbon degradation, hydrocarbon degradation, aromatic hydrocarbon degradation, aromatic compound degradation, cellulolysis, xylanolysis, chitinolysis, fermentation, methylotrophy, and methanol oxidation. Dark hydrogen oxidation was relatively more abundant under W2Q1 and W1Q2, whereas chitinolysis was relatively more abundant under W2Q1 and W3Q2. Predicted nitrogen cycle-related functions included nitrogen fixation, nitrogen respiration, nitrate respiration, and nitrate reduction. In addition, functions associated with manganese oxidation, dark iron oxidation, phototrophy, and photoheterotrophy were also detected across the treatments. Overall, the substrate bacterial communities under all treatments were dominated by functions related to heterotrophic metabolism and organic matter degradation, while also exhibiting the potential to participate in nitrogen transformation, hydrocarbon degradation, and iron and manganese cycling.

## 3. Discussion

### 3.1. The Lower Irrigation Limit Shapes Root Plasticity by Regulating the Substrate Microenvironment

Coir substrate has a high water-holding capacity and porosity, and its root-zone environment is determined not only by irrigation volume but also by the combined effects of the lower irrigation limit and drip irrigation flow rate [22,23,24]. The lower irrigation limit primarily influences the mean water status of the coir substrate and its temporal variation [24], whereas drip irrigation flow rate mainly affects the rate of water movement after irrigation, the shape of the wetting front, and the spatial distribution of moisture within the substrate [22]. In the present study, the 70% lower irrigation limit treatment maintained a higher and relatively stable substrate water content, whereas the 30% lower irrigation limit treatment exhibited greater moisture fluctuation, indicating that the lower irrigation limit primarily altered the temporal heterogeneity of the root-zone moisture environment. Although the different drip irrigation flow rates did not change the overall pattern of periodic fluctuation in substrate water content, they may have further regulated the root-zone environment by affecting post-irrigation water redistribution and the extent of localized wetting. Large wetting–drying fluctuations may repeatedly expose roots and microorganisms to dehydration–rewetting cycles. During dehydration, water-film connectivity and the diffusion of soluble substrates decline, while osmotic stress intensifies. Rewetting may subsequently generate short-term resource pulses that promote the recovery and growth of drought-tolerant or rapidly responding microbial taxa. A 70% lower irrigation limit can alleviate periodic water deficits [24] and provide a relatively stable environment for maintaining root nutrient uptake and metabolic activity [25]. However, continuously high water contents may also reduce air-filled porosity and restrict oxygen diffusion. Consequently, the ecological effects of increasing substrate water content may not necessarily increase linearly [26,27]. The slightly lower substrate temperatures observed under the higher drip irrigation flow rate treatments may be associated with increased heat capacity and evaporative cooling. Nevertheless, because substrate temperature was also strongly affected by greenhouse climatic conditions and seasonal variation, the direct effect of drip irrigation flow rate requires further verification.

Tomato roots exhibited pronounced morphological and physiological plasticity in response to the different irrigation combinations. As the lower irrigation limit increased, total root length, root surface area, and the number of root tips generally increased, whereas mean root diameter decreased. These responses indicate that a wetter substrate environment promoted fine-root elongation, branching, and expansion of the absorptive surface. Fine roots and root tips are not only major sites of water and nutrient uptake but also principal interfaces for root exudation and microbial colonization. Their proliferation may therefore expand the spatial extent of plant influence on substrate microbial communities [28,29].

The statistical responses of individual root traits to the irrigation factors differed markedly. Two-way ANOVA showed that the lower irrigation limit significantly affected all root traits, whereas drip irrigation flow rate and its interaction with the lower irrigation limit significantly affected only root volume. Based on the treatment means, the combination of a high lower irrigation limit and low drip irrigation flow rate resulted in greater total root length, root surface area, and number of root tips, whereas the combination of a moderate lower irrigation limit and high drip irrigation flow rate produced the greatest root volume and relatively high root activity. However, except for root volume, neither drip irrigation flow rate nor its interaction with the lower irrigation limit had significant effects on the other root traits. These findings suggest that root responses depend not only on mean substrate water content but also on the balance among the rate of water supply, the extent of the wetted zone, nutrient transport, and root-zone aeration. A lower flow rate may favor gradual water diffusion and spatial expansion of the root system, whereas a higher flow rate may increase the short-term integrity of water and nutrient supply. Under relatively wet conditions, however, rapid water application may also create locally oversaturated zones and restrict root respiration. Thus, 50% and 70% lower irrigation limits were generally beneficial for root growth, but the highest substrate water content did not produce the optimum response for every root trait.

### 3.2. Substrate Environmental Filtering and Plant Selection Jointly Drive Bacterial Community Reassembly

The composition of rhizosphere bacterial communities is commonly shaped by environmental filtering imposed by soil or substrate conditions. Among these factors, moisture status, pH, electrical conductivity, and nutrient availability, including nitrogen, phosphorus, potassium, and organic carbon, may all play important roles. By contrast, the diversity and temporal dynamics of active bacterial communities are influenced not only by environmental filtering but also more directly by rhizosphere carbon inputs, root exudation, and other forms of plant-mediated selection [29,30,31]. The effects of irrigation on bacterial communities were therefore expressed primarily as shifts in community composition rather than as linear changes in species richness. In the present study, the Chao1 index varied only slightly among treatments, whereas PCoA and PERMANOVA revealed clear separation in community structure. This pattern suggests that the coir substrate may harbor a relatively stable microbial species pool, while irrigation conditions primarily reorganize the community by altering the ecological dominance of different taxa. Changes in water availability can simultaneously affect water-film connectivity, substrate diffusion, oxygen supply, osmotic stress, and root physiology [32,33,34]. Together, these factors impose environmental filters on bacteria with different ecological strategies [35]. Two-way ANOVA showed that the Chao1 index was not significantly affected by the lower irrigation limit, drip irrigation flow rate, or their interaction, whereas the Simpson dominance index (D) and Shannon index both exhibited significant lower irrigation limit × drip irrigation flow rate interaction effects. This indicates that the effect of drip irrigation flow rate on bacterial community α-diversity depended on the specific lower irrigation limit, rather than exhibiting a consistent unidirectional response to flow rate. Meanwhile, the lack of a significant response in the Chao1 index suggests that different irrigation combinations had relatively limited effects on bacterial richness, whereas changes in community diversity were more closely associated with shifts in the relative abundance distribution of different bacterial taxa. A lower flow rate may maintain greater microscale environmental heterogeneity, allowing bacteria with contrasting resource-use strategies to coexist. By contrast, a higher flow rate may create localized zones of concentrated resources and thereby favor rapidly growing taxa.

The differentially abundant taxa further reflected ecological selection under contrasting moisture regimes. Under the lower irrigation limit, the enrichment of Streptomycetaceae and other actinomycete-associated taxa was consistent with previous observations that drought conditions select for Actinobacteria and *Streptomyces*. This response may be associated with desiccation-resistant structures, tolerance to osmotic stress, and irrigation-induced changes in root metabolism and plant immune status [36,37,38]. In contrast, the increased abundance of Bacteroidota and Flavobacteriales may be related to the degradation of plant-derived polysaccharides and organic components of the coir substrate [39]. The enrichment of Bacillota, *Bacillus*, *Neobacillus*, and *Priestia* under the combination of a 50% lower irrigation limit and a low flow rate may reflect the adaptation of spore-forming bacteria to moderate wetting–drying fluctuation [37]. However, these taxa did not attain their highest abundance under W1Q1, indicating that drought tolerance was not the sole driver of their distribution. Rhizosphere resource availability, oxygen conditions, and interspecific competition may also have contributed to their selection. The combination of a 50% lower irrigation limit and a high flow rate enriched Pseudomonadota, Burkholderiales, *Cellvibrio*, and *Devosia*. These bacteria generally exhibit strong responses to substrate availability and a high capacity for rhizosphere colonization. Their enrichment may therefore be associated with greater root activity, a more abundant supply of available carbon, and enhanced water-mediated substrate diffusion [30,37]. The enrichment of *Pseudomonas* under the combination of a 70% lower irrigation limit and a high flow rate further suggests that this genus possesses a strong competitive advantage in relatively moist environments. However, *Pseudomonas* includes plant growth-promoting bacteria, antagonists, saprotrophs, and potential pathogens, and the V3–V4 region of the 16S rRNA gene cannot reliably distinguish functional differences at the strain level. Consequently, the enrichment of specific taxa indicates a shift in ecological dominance but does not directly demonstrate enhanced plant growth promotion, disease suppression, or pathogenicity. Overall, the lower irrigation limit may determine the general direction of environmental filtering during community assembly, whereas drip irrigation flow rate may exert further regulatory effects by altering microscale heterogeneity within the root zone.

### 3.3. Nonlinear Response of Bacterial Association Networks and Predicted Function to Irrigation Combination

The effects of moisture variation on microbial networks are inherently context-dependent and nonlinear [40]. In the present study, the co-occurrence networks showed that microbial association patterns did not respond linearly to increasing lower irrigation limits. The combination of a 50% lower irrigation limit and a high flow rate produced a network with greater connectivity and modularity, whereas connectivity declined under the combination of a 70% lower irrigation limit and a high flow rate. This pattern suggests that moderately wet conditions may simultaneously sustain substrate diffusion, rhizosphere carbon inputs, oxygen availability, and microscale heterogeneity, thereby providing a greater range of ecological niches for bacteria with different ecological strategies. By contrast, persistently high substrate water content may reduce certain microenvironmental differences or promote the dominance of a limited number of moisture-adapted taxa, concurrently weakening some cross-taxon associations. It should be emphasized that co-occurrence networks represent statistical association among taxon abundances. Correlation within these networks cannot be interpreted directly as cooperation or competition, and network connectivity and modularity alone do not demonstrate enhanced ecological stability [41,42,43]. The present results should therefore be regarded as exploratory evidence that different irrigation combinations altered potential bacterial association patterns. FAPROTAX prediction further indicated that the potential functional profiles of the bacterial communities responded differently to the irrigation treatments. Chemoheterotrophy and aerobic chemoheterotrophy were dominant under all treatments, together with predicted capacities for the degradation of cellulose, xylan, chitin, and aromatic compounds. This functional pattern is consistent with the abundance of plant-derived organic matter in coir and the continuous input of plant-derived carbon into the rhizosphere [44]. Water supply may regulate carbon-utilization function by altering the dissolution and diffusion of organic substrates, while also influencing fermentation, nitrate reduction, and other nitrogen-transformation processes through changes in oxygen diffusion and redox conditions. Although the relative changes in predicted carbon-degradation and nitrogen-cycling function were broadly consistent with the reassembly of dominant bacterial taxa, these results are insufficient to demonstrate corresponding changes in actual carbon and nitrogen transformation rates.

## 4. Materials and Methods

### 4.1. Experimental Site and Materials

**Figure 8:**
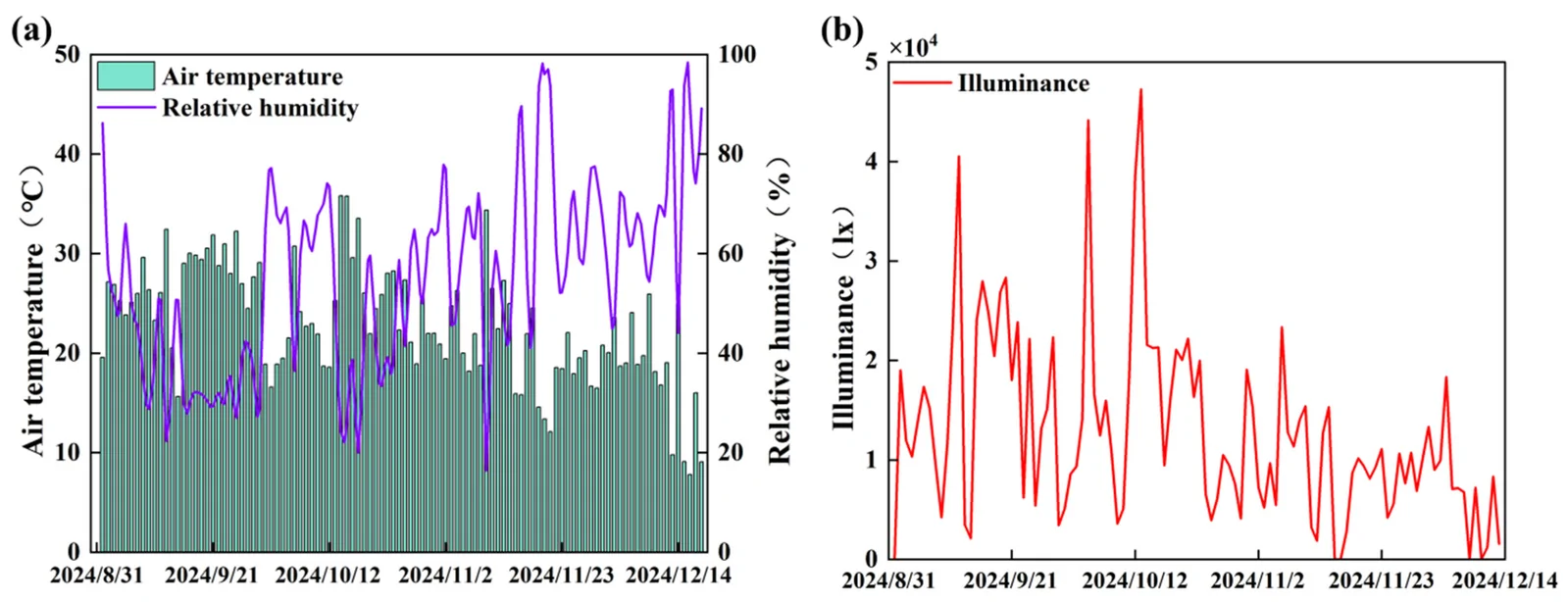
Temporal variation in daily mean greenhouse environmental conditions during the experimental period. (**a**) Air temperature (bars) and relative humidity (line); (**b**) illuminance.

The experiment was conducted at the Yecheng Gobi Experimental Base in Kashgar, southern Xinjiang, China. The site (37.807° N, 77.538° E; 1397 m above sea level) is located in the traditional zone between the northern foothills of the Karakoram Mountains and the southwestern margin of the Taklamakan Desert. The region has a typical temperate continental arid climate, with a mean annual temperature of 11.3 °C, annual precipitation of 67 mm, and a frost-free period exceeding 230 days. The tomato cultivar ‘Provence’ (*Solanum lycopersicum* L.) was used in the experiment, and the seedlings were provided by Shanghai Sunqiao Yijia Agricultural Technology Co., Ltd., Shanghai, China. The coir substrate consisted of a 1:1 mixture of fine coir particles (0–6 mm) and coarse coir particles (10–20 mm). The coir substrate was not sterilized before use and was obtained from the same production batch, thoroughly mixed, and evenly distributed among experimental units to ensure comparable initial conditions across treatments. Its main physicochemical properties were a water-holding capacity of 42.12% and a bulk density of 0.128 g·cm^−3^. The container water-holding capacity of the substrate, determined using the saturation–free drainage method, was defined as 100% water-holding capacity [45]. Uniform seedlings at the five-leaf-one-heart stage were transplanted on 25 August 2024, and the experiment ended on 15 January 2025. Air temperature, relative humidity (RH), and illuminance inside the greenhouse were continuously monitored throughout the experimental period, and the temporal variation in these environmental variables is shown in Figure 8.

### 4.2. Experimental Design and Treatments

The experiment employed a 3 × 2 two-factor randomized block design. The lower irrigation limits were set at 30%, 50%, and 70% of substrate water-holding capacity and were designated W1, W2, and W3, respectively. These levels were selected to establish contrasting low, intermediate, and high lower irrigation limits, with reference to previous studies in which graded lower irrigation limits based on field capacity (FC), available water, or substrate water-holding capacity were used to regulate root-zone water conditions in tomato cultivation [24,46,47]. Drip irrigation flow rates were set at 1 and 2 L·h^−1^ and were designated Q1 and Q2, respectively, resulting in six treatments. Each treatment included three independent cultivation troughs as biological replicates, yielding a total of 18 experimental units. The treatments were randomly arranged within blocks in the greenhouse, and the detailed experimental design is presented in Table 3. Each experimental unit was equipped with an independent irrigation lateral, a control valve, and a pressure-compensating dripline with the corresponding flow rate. The emitter spacing was 20 cm. Tomatoes were cultivated in sunken troughs measuring 30 cm in both width and depth. Geotextile fabric was laid at the bottom of each trough to prevent moisture exchange with the underlying soil. Plants were arranged in staggered rows, with a row length of 12 m, a row spacing of 1.5 m, and a plant spacing of 20 cm, resulting in 60 plants per row. Irrigation was triggered according to substrate volumetric water content. Irrigation began when the water content declined to the designated lower irrigation limit and stopped when it reached 90% of substrate water-holding capacity. The substrate was thoroughly drip-irrigated on the day of transplanting to ensure seedling establishment and survival. The irrigation treatments were initiated on the following day, with irrigation subsequently triggered according to the designated lower irrigation limits. Tomato cultivation was managed by Shanghai Sunqiao Yijia Agricultural Technology Co., Ltd. (Shanghai, China). A locally used commercial water-soluble solid fertilizer was applied throughout the experimental period following the company’s recommended stage-specific fertigation program for greenhouse tomato production. According to the formulation information provided by the company, the fertilizers contained 19–21% N, 3–4% P, 18–36% K, 10–20% Ca, and 4–6% Mg on a mass basis.

### 4.3. Measurements

#### 4.3.1. Daily Mean Substrate Water Content and Temperature

Substrate volumetric water content and temperature were automatically monitored using moisture–temperature sensors (TEROS 12, METER Group, Pullman, WA, USA) installed at a depth of 10 cm. Data were recorded at 30 min intervals and transmitted in real time to the data acquisition platform. During the experimental period, all monitoring data were cleaned and calibrated, and daily mean values were calculated for subsequent analyses.

#### 4.3.2. Root Traits and Root Activity

Intact root systems were collected at the fruit maturity stage before the tomato plants were removed. Three healthy plants were randomly selected from each treatment as biological replicates. After being thoroughly washed, the roots were placed in a root tray containing a 0.5 cm deep layer of water to ensure complete spreading. Root images were then acquired at a resolution of 300 dpi using a root scanner (Epson, Suwa, Japan). Root morphological traits, including total root length (Tl), root surface area (Rs), root volume (Rv), mean root diameter, and number of root tips, were analyzed using WinRHIZO Pro Vision 5.0 software (Regent Instruments Inc., Quebec City, QC, Canada) [48]. Root activity was determined using the triphenyl tetrazolium chloride (TTC) reduction method [49]. Fresh root samples (0.5 g) were immersed in a mixture of 10 mL of 0.4% TTC solution (Shanghai Yuanye Bio-Technology Co., Ltd., Shanghai, China) and 10 mL of phosphate buffer (pH 7.0; Macklin Biochemical Co., Ltd., Shanghai, China) and incubated at 37 °C in the dark for 3 h. The reaction was terminated by adding 2 mL of 1 mol·L^−1^ H_2_SO_4_ (Sinopharm Chemical Reagent Co., Ltd., Shanghai, China). The resulting triphenyl formazan (TPF) was extracted with ethyl acetate (Sinopharm Chemical Reagent Co., Ltd., Shanghai, China), and absorbance was measured at 485 nm using a UV-2600 spectrophotometer (Shimadzu Corporation, Kyoto, Japan). Root activity was calculated from a standard curve and expressed as mg·g^−1^·h^−1^.

#### 4.3.3. Substrate Sample Collection and Illumina MiSeq Sequencing

Root-zone coir substrate samples were collected at the tomato fruit maturity stage. Three replicates were collected from each treatment, yielding a total of 18 samples. Immediately after collection, the samples were placed in sterile polyethylene bags, stored with ice packs, and rapidly transported to the laboratory. They were then frozen at −80 °C until subsequent analyses of substrate microbial community structure and functional potential. The 16S rRNA gene was amplified using the primer pair 338F/806R. The purified PCR products were sequenced on an Illumina MiSeq platform (Illumina Inc., San Diego, CA, USA) by Majorbio Bio-Pharm Technology Co., Ltd. (Shanghai, China).

### 4.4. Statistical Analysis

Data were organized using Microsoft Excel 2024 (Microsoft Corporation, Redmond, WA, USA) and statistically analyzed using SPSS 27.0 (IBM Corp., Armonk, NY, USA). For root traits and related variables, one-way analysis of variance (one-way ANOVA) was used to compare differences among irrigation treatments. When significant treatment effects were detected, means were compared using Duncan’s multiple range test (α = 0.05). In addition, two-way analysis of variance (two-way ANOVA) was performed to evaluate the effects of the lower irrigation limit (W), drip irrigation flow rate (Q), and their interaction (W × Q) on root traits and bacterial α-diversity indices. Statistical significance was set at *p* < 0.05. Bacterial α-diversity was analyzed using the I-Sanger Cloud Platform provided by Majorbio Bio-Pharm Technology Co., Ltd. (Shanghai, China), and principal coordinate analysis (PCoA) was performed based on Bray–Curtis distances. Bacterial community diversity, community composition, and functional profiles were analyzed using QIIME 2 (QIIME 2 Development Team, USA) and FAPROTAX (FAPROTAX Development Team, USA), as appropriate. Figures were generated using Origin 2022 (OriginLab Corporation, Northampton, MA, USA). For bacterial co-occurrence network analysis, the top 100 ASVs ranked by relative abundance were selected as candidate nodes. Associations among ASVs were evaluated using Spearman’s correlation analysis, and correlations with |r| ≥ 0.6 and *p* < 0.05 were retained for network construction. The resulting networks were visualized using Gephi v. 0.10.0 (Gephi Consortium, Paris, France), and figures were subsequently arranged and combined using Microsoft PowerPoint (Microsoft Corporation, Redmond, WA, USA).

## 5. Conclusions

The lower irrigation limit and drip irrigation flow rate jointly regulated the root-zone moisture environment of the coconut coir substrate under Gobi greenhouse conditions and exerted differential effects on tomato root growth and bacterial communities. The lower irrigation limit primarily determined the substrate moisture level and its temporal fluctuation and was also the main factor affecting tomato root traits, whereas drip irrigation flow rate and its interaction with the lower irrigation limit significantly affected only root volume among the measured root traits. Higher lower irrigation limits generally promoted root elongation, expansion of the absorptive surface area, and root activity. However, individual root traits responded differently to irrigation combination, indicating that no single irrigation regime simultaneously optimized root morphology, physiological activity, and the root-zone microbial environment. Different irrigation treatments significantly reshaped bacterial community structure, whereas bacterial richness showed relatively limited variation. Significant interaction between the lower irrigation limit and drip irrigation flow rate was observed for the Simpson dominance index (D) and Shannon index, indicating that irrigation regulation was reflected mainly in shifts in community composition, relative abundance distribution, and diversity patterns. Co-occurrence network analysis and functional prediction further revealed nonlinear responses of bacterial association patterns and potential metabolic functions to different irrigation combinations. Overall, different irrigation combinations exhibited distinct advantages in regulating root growth and the root-zone microbial environment. Therefore, the lower irrigation limit and drip irrigation flow rate should be appropriately matched according to specific production objectives and root-zone management goals in Gobi greenhouse tomato cultivation using coconut coir substrate.

## Figures and Tables

**Table 1:** Summary of two-way ANOVA statistics for root traits and bacterial α-diversity indices.

Parameters	W (F)	W (P)	Q (F)	Q (P)	W × Q (F)	W × Q (P)
Ra	5.439	0.021	1.07	0.321	0.418	0.668
Rv	84.805	<0.001	109.068	<0.001	21.694	<0.001
Tl	26.036	<0.001	0.045	0.835	0.373	0.697
Ad	16.398	<0.001	0.11	0.746	0.31	0.739
Rs	18.811	<0.001	1.145	0.306	0.564	0.584
Rt	34.19	<0.001	2.843	0.118	1.067	0.374
Chao1	0.097	0.909	1.821	0.202	3.024	0.086
Simpson	1.325	0.302	1.171	0.301	7.553	0.008
Shannon	0.296	0.749	3.844	0.074	9.235	0.004

Ra, root activity; Rv, root volume; Tl, total root length; Ad, average root diameter; Rs, root surface area; Rt, root tip number. W, lower irrigation limit; Q, drip irrigation flow rate; W × Q, interaction between lower irrigation limit and drip irrigation flow rate. Statistical significance was determined at *p* < 0.05.

**Table 2:** Topological properties of the bacterial co-occurrence networks.

Property	Treatment					
	W1Q1	W2Q1	W3Q1	W1Q2	W2Q2	W3Q2
Nodes	99	99	99	99	100	100
Edges	1524	1550	1547	1543	1622	1447
Average degree	30.79	31.63	31.25	31.17	32.44	28.94
Graph deity	0.314	0.326	0.319	0.318	0.328	0.292
Modularity	0.582	0.624	0.643	0.649	0.662	0.658
Positive correlation %	53.35	50.32	51.45	55.35	53.76	51.76
Negative correlation %	46.65	49.68	48.55	44.65	46.24	48.24

**Table 3:** Experimental design and treatment combination.

Treatment	Drip Irrigation Flow Rate (L·h^−1^)	Lower Irrigation Limit		Upper Irrigation Limit	
		Decrease to Field Capacity (%FC)	Volumetric Water Content (%)	Increase in Field Capacity (%)	Volumetric Water Content (%)
W1Q1	1	30	12	90	36
W2Q1		50	20		
W3Q1		70	28		
W1Q2	2	30	12		
W2Q2		50	20		
W3Q2		70	28		

## Data Availability

All generated or analyzed data that are presented in the paper and all figures are available from the corresponding author upon request.
